# Metastasizing ameloblastoma: a case report

**DOI:** 10.1186/s13256-025-05568-6

**Published:** 2025-11-03

**Authors:** Rohan Shetty, Rohan Thomas Mathew, Rohit Singh

**Affiliations:** Department of Surgical Oncology, Zulekha Yenepoya Institute of Oncology, Yenepoya (Deemed to Be University), Mangalore, Karnataka 575018 India

**Keywords:** Ameloblastoma, Metastasizing ameloblastoma, Pulmonary metastasis

## Abstract

**Background:**

Metastasizing ameloblastoma is a rare, distinct entity under the World Health Organization classification. It is a unique tumor in which metastasis can occur but remains benign. Incidence is considered to be around 2% of reported cases of ameloblastoma, but it could be much lower.

**Case presentation:**

A 30-year-old South Indian woman of Dravidian ethnicity presented in the year 2020 with swelling over the right side of the face. She had a history of recurrent ameloblastoma on the left side of the mandible, for which she underwent multiple surgeries in the past 8 years. A right hemimandibulectomy with a patient-specific implant was performed, and the patient recovered well. She developed hypersensitivity to the implant, which was subsequently removed in 2021. In 2024, she developed a persistent cough with blood-tinged sputum. This did not subside with conservative management and hence was further evaluated. Imaging revealed multiple small, well-defined nodules in both lungs and a branching tubular hypodense lesion in the right parahilar region. Bronchoscopy-guided biopsy was done on the lesion. This was reported as metastatic ameloblastoma. The case was discussed in the multidisciplinary tumor board. It was decided to start the patient on a multikinase inhibitor. However, the patient refused further treatment. The patient is reported to be symptomatically better.

**Conclusion:**

Ameloblastoma is a benign but locally aggressive odontogenic tumor that rarely metastasizes. We report a case with multiple local recurrences over 12 years, currently with pulmonary metastasis. Pulmonary metastasis may be indolent and hence could be misdiagnosed or missed altogether. The degree of suspicion should be higher for patients with recurrences.

**Supplementary Information:**

The online version contains supplementary material available at 10.1186/s13256-025-05568-6.

## Background

Metastasizing ameloblastoma (METAM) has been a distinct entity under the World Health Organization (WHO) Classification of the Odontogenic Tumours from the 3rd edition, which was published in 2005. This is histologically benign even at the site of metastasis. This differs from ameloblastic carcinoma, which has ameloblastoma features and carcinoma features. This separation continued in the 4th and the recent 5th editions, published in 2022 [1]. The reason for the separation is the clinical behavior and histopathological features. METAM has an indolent course, preserving the benign histology even at the location of metastasis, whereas ameloblastic carcinoma is malignant, with the features and histology of carcinoma. The common site of metastasis of the former is pulmonary metastasis, followed by the lymph nodes. The incidence is thought to be around 2% of reported cases of ameloblastoma, but recent reports suggest that it is much lower [2].

The exact mechanism of metastasis is unknown. Incomplete resection, tumor spillage, multiple surgeries, direct tumor seeding, and hematogenous spread are all suggested. The extremely low number of reported cases makes it difficult to understand the pathogenesis of the disease. We present a case of a young female patient of South Indian descent who had multiple recurrences of ameloblastoma in the mandible over 12 years before being diagnosed with pulmonary metastasis.

## Case presentation

**Fig. 1:**
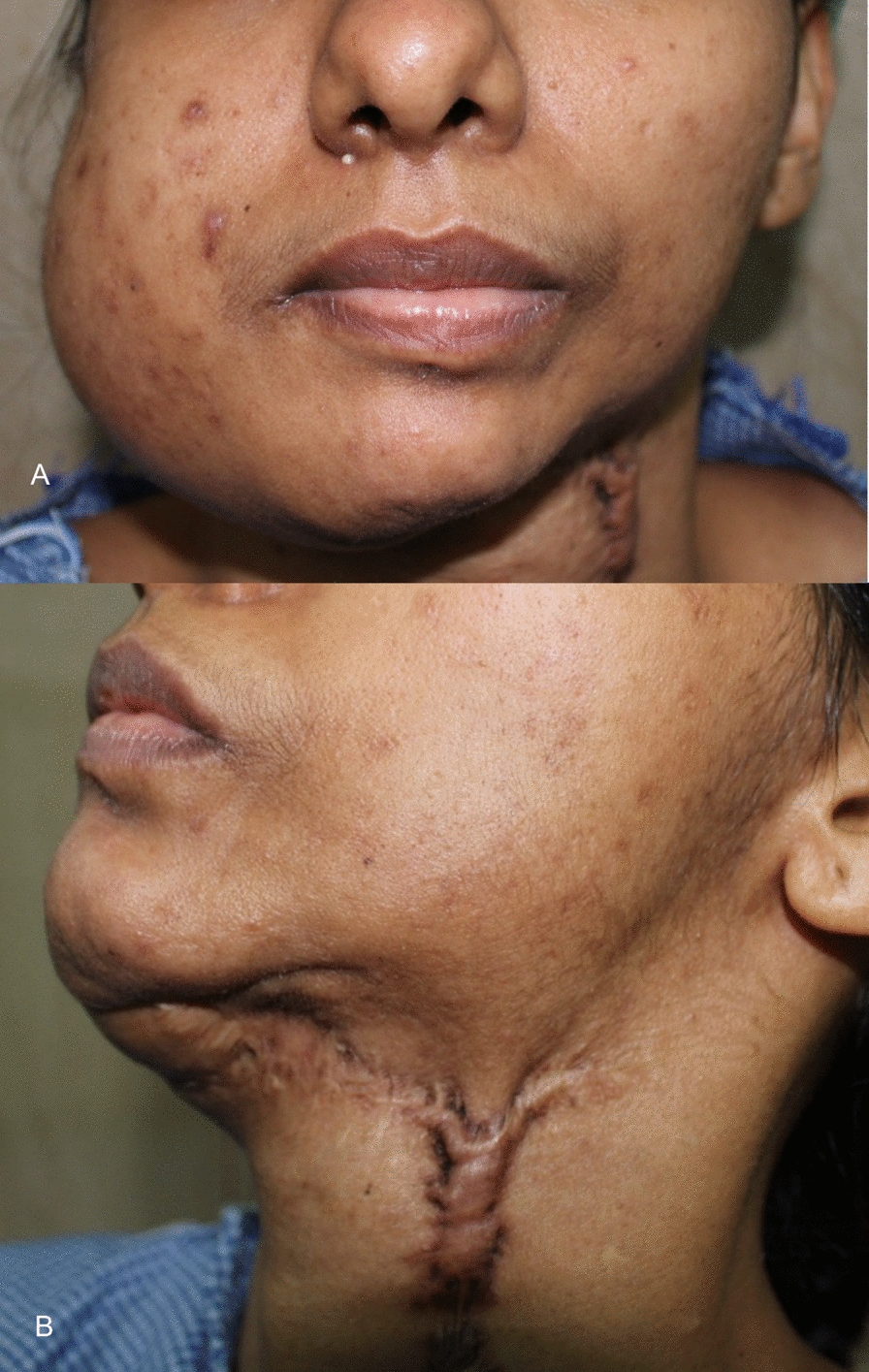
**A** Swelling over the right side, anterior view. **B** Scar from the previous surgeries on the left side

A 30-year-old South Indian woman of Dravidian ethnicity presented to our department in 2020 with a gross swelling over the right side of her face. She had a long history of recurrent ameloblastoma occurring on the left side of the mandible initially in 2012, for which enucleation and plating were done. She developed a recurrent ameloblastoma in the left body of the mandible in 2014 and underwent resection and reconstruction with a free fibula flap. The histopathology was follicular ameloblastoma. After about 7 months, the hardware got infected, and the hardware and the flap had to be removed. In 2016, she underwent secondary reconstruction with the free fibula flap harvested from the contralateral side. During the surgery, suspicious tissue was found and sent for histopathological examination. It was reported as a recurrent ameloblastoma. She was disease-free until 2020, when she presented with significant swelling over the right side of her face. (Fig. 1a, b) The scars from the previous surgeries were visible on the left side.

**Fig. 2:**
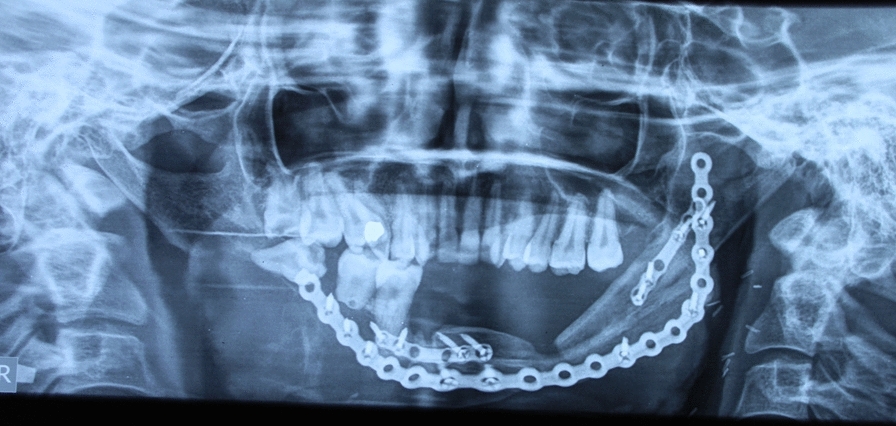
Orthopantomogram showing a radiolucent lesion on the left side and a fibula flap with recon plates on the left side

Imaging revealed an extensive radiolucent lesion involving the mandible’s right condyle, ramus, and body. The left side showed the fibula flap with the recon plates. (Fig. 2) Clinically, this was determined to be a recurrence of the ameloblastoma, and surgical excision was planned. A right hemimandibulectomy was performed, and the defect was reconstructed using a patient-specific implant and primary closure. No flaps were used during this surgery. Postoperative course was uneventful. Final histopathology examination of the resected specimen was reported as ameloblastoma. The report showed that the specimen had solid and cystic components. The tumor cells are arranged in follicular and plexiform patterns with acanthotic areas. The tumor was found to be invading the surrounding skeletal muscles. BRAF mutation was tested on this specimen, but was reported to be negative.

However, 6 months later, the patient developed a hypersensitivity reaction to the implant, leading to extraoral wound dehiscence. This was managed conservatively, and 4 months later, intraoral wound dehiscence also began, necessitating implant removal under general anesthesia with wound debridement and primary closure. The patient and her family refused further surgeries or any corrections, and the patient was placed on regular follow-up.

In May 2024, the patient reported to our department with complaints of a persistent cough with occasional blood-tinged sputum, which had been ongoing for the past 3 months. The patient also reported fatigue, weight loss, and decreased appetite. Reduced breath sounds were noted on auscultation, but supraclavicular or cervical nodes were not palpable. She was first managed conservatively. Intraoral examination was routine, and there were no signs of local recurrence. Since the symptoms did not subside with conservative measures, she was evaluated further.

High-resolution chest computed tomography (CT) was performed, which revealed multiple small, well-defined pulmonary nodules in both lungs, involving the upper and lower lobes, as well as the right middle lobe, with the largest measuring 10 mm. A branching tubular hypodense lesion was also noted in the right parahilar region, specifically around the right middle lobe. The right middle lobe bronchus was not seen separately. These findings were suggestive of metastasis, with differential diagnoses including tuberculosis and primary lung malignancy. The sputum test was negative for tuberculosis, and she did not have any risk factors for primary lung cancer.

The patient underwent further evaluation with a bronchoscopy.

**Fig. 3:**
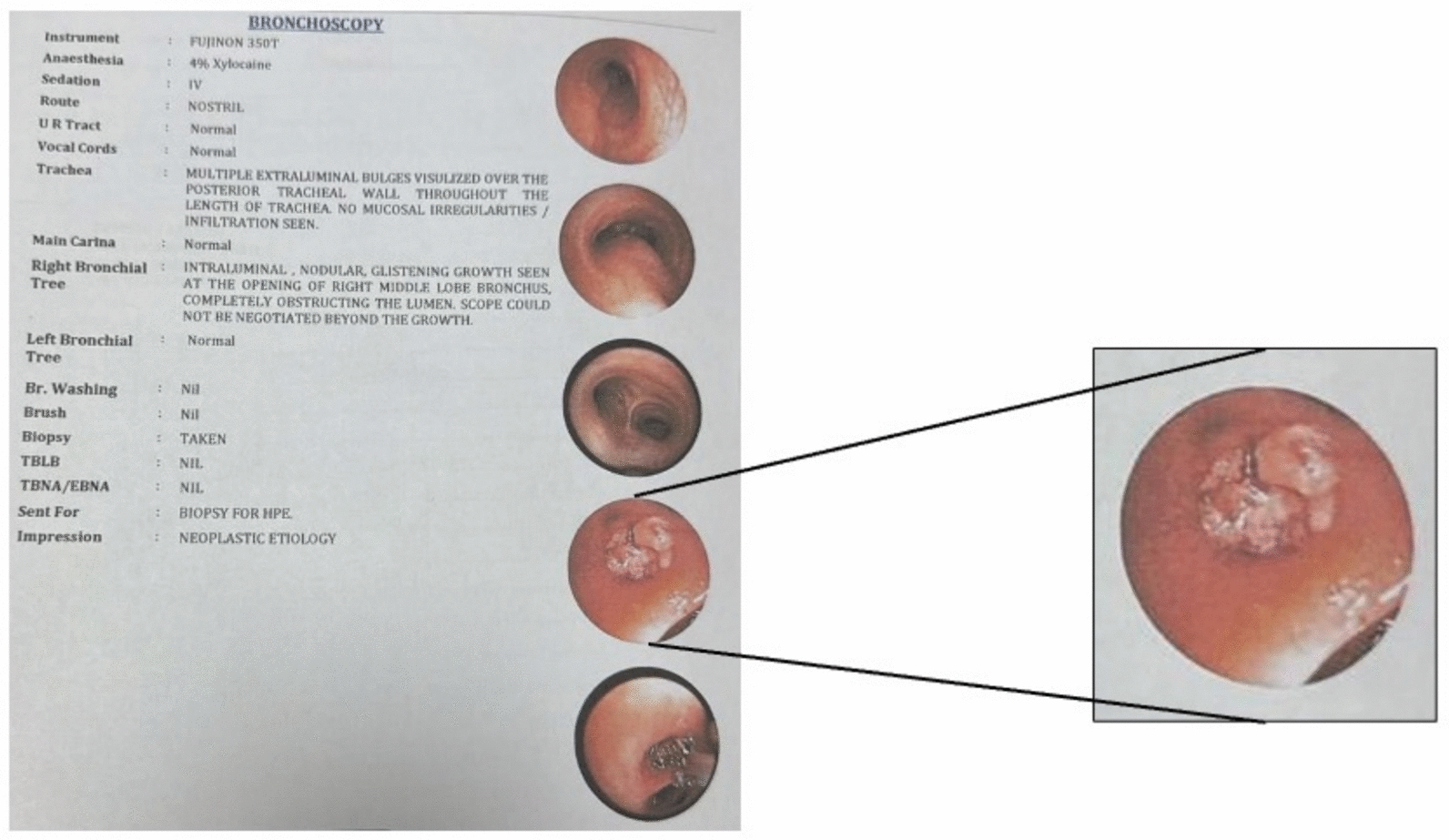
Bronchoscopy report showing growth in the right bronchial tree

It identified multiple extraluminal bulges visualized over the posterior tracheal wall, throughout the trachea’s length, with no mucosal irregularity. However, the right bronchial tree showed an intraluminal nodular glistening growth at the opening of the right middle lobe bronchus, completely obstructing the lumen. The scope could not be advanced beyond this point. (Fig. 3).

**Fig. 4:**
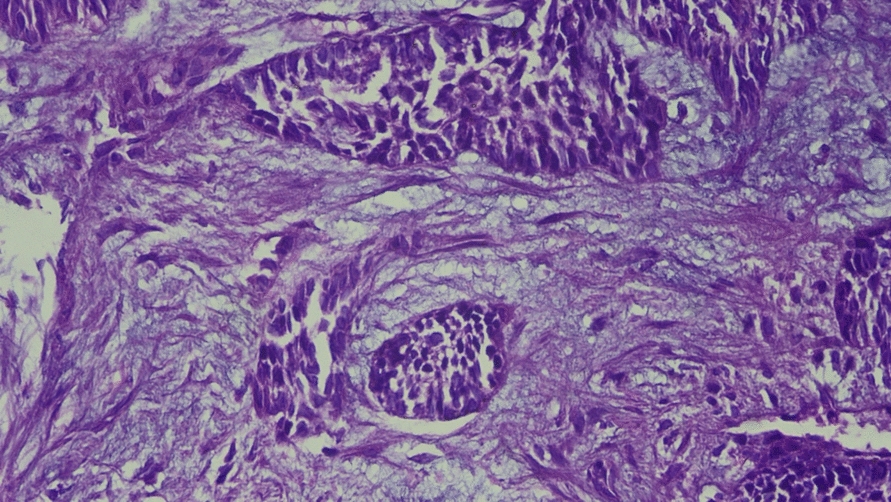
Hematoxylin and eosin staining (20×) showing neoplastic cells in islands around a loose network resembling stellate reticulum with myxoid stroma

A biopsy was taken from this lesion. The specimen from the right middle lobe of the lung showed multiple fragments of tissue with a neoplasm composed of tumor cells arranged in cords and islands, containing intraluminal basophilic material. These neoplastic cells exhibited moderate anisonucleosis with eosinophilic cytoplasm and were arranged to form a loose network resembling stellate reticulum. Foci of squamoid nests were noted, and mitoses were sparse. Intervening areas of myxoid matrix were observed. One of the fragments showed respiratory epithelium along with mature cartilage. No marked atypia, increased mitoses, or necrosis was noted. (Fig. 4) These findings suggested metastasizing ameloblastoma (METAM). Further subtyping was not possible from the biopsy specimen.

The multidisciplinary tumor board discussed the case, and it was decided to start the patient on multikinase inhibitors while testing the specimen for any actionable mutation. However, the patient refused further testing on the specimen and further treatment after being informed of the treatment and the outcomes. With this available evidence, we advised our patient to take sorafenib, but she also refused any treatment.

The patient’s family was last contacted in March 2025, who reported that the patient was comfortable and with no progression of the symptoms. However, they declined further imaging or treatment. The clinical timeline is presented in Table 1.

**Table 1:** Clinical timeline

Month/year	Event
2012	First diagnosis of ameloblastoma (left mandible); treated with enucleation and plating
April 2014	Recurrence in the left mandibular body; resection and free fibula flap reconstruction performed
December 2014	Hardware and flap removal due to infection
2016	Secondary reconstruction with contralateral free fibula flap; intraoperative biopsy confirmed recurrent ameloblastoma
Disease free (2016–2020)	No clinical or radiological evidence of recurrence
2020	Presented with swelling over the right side of the face; imaging showed lesions in right condyle, ramus, and body. Right hemimandibulectomy performed with patient-specific implant reconstruction Histopathology confirmed ameloblastoma with solid/cystic areas, follicular and plexiform patterns, and muscle invasion
2021 (~6 months postoperative)	Developed hypersensitivity to implant with extraoral wound dehiscence; managed conservatively
2021 (~10 months postoperative)	Intraoral wound dehiscence; implant removed under general anesthesia with wound debridement and closure
2021–2023	Patient declined further surgery; placed on routine follow-up
February 2024	Developed persistent cough, hemoptysis, fatigue, weight loss, and anorexia
May 2024	High-resolution CT revealed multiple pulmonary nodules in both lungs and branching lesion near right middle lobe bronchus, suggestive of metastasis Bronchoscopy: intraluminal nodular glistening mass completely obstructing right middle lobe bronchus. Biopsy taken Biopsy: histopathology consistent with metastasizing ameloblastoma (METAM) with adenoid features
May 2024	Tumor board recommended BRAF mutation testing and targeted therapy. Patient refused testing and treatment
March 2025	Last contact with patient’s family: patient reportedly stable, no symptom progression, but declined imaging or further treatment

## Discussion

Ameloblastoma is a rare, benign odontogenic tumor that is locally aggressive and has a high chance of local recurrence. It constitutes less than 1% of all the tumors and cysts of the jawbones. It affects both genders and is predominantly seen in the lower jaw. It typically presents as a painless swelling of the posterior mandible or ascending ramus. Inadequate removal will almost always result in local recurrence. Segmental resection with reasonable margins is the accepted treatment [2]. Although the incidence of metastasizing ameloblastoma is infrequent, one of the first described reports of a pulmonary metastasis from a jawbone adamantinoma is by Vorzimer and Perla, published in 1932. The authors report that pulmonary metastasis has no malignant features, but they suspect that the etiology is the aspiration of the primary tumor cells and direct seeding [3]. From the 3rd edition of the WHO classification of tumors, METAM is considered under benign epithelial odontogenic tumors, whereas ameloblastic carcinoma is considered under odontogenic carcinomas. Earlier classifications had clubbed them together as malignant ameloblastoma. This has led to confusion and does not reflect the clinical or histological features. This change highlights its benign and possibly indolent nature [4].

METAM is extremely rare, and according to a recent systematic review, only 661 cases have been reported. The most common metastasis is to the lungs, which accounts for approximately 72% of all reported cases. Other sites are the lymph nodes, bone, kidney, pelvis, and the brain [5]. There was a case report that reported metastasis to the chest wall and caused pleural effusion [6]. The risk factors for metastasis are said to be multiple surgeries for the primary tumor and a lower age of first occurrence of the disease. Both these risk factors were present in the patient reported to us, who had a history of multiple recurrences in the last 12 years, and who had undergone the first surgery at 22 years old. No factors can confidently predict metastasis because of the extremely low number of reported cases. It is also difficult to standardize the treatment due to this.

Different management methods are described, ranging from no treatment to surgical resection, chemotherapy, radiotherapy, and targeted therapy. Surgical resection is preferred in resectable cases since the primary tumor is best treated with surgical resection. This has also been reported to have the highest cure rate [2]. However, there are also case reports in which the patients have not undergone any treatment and have lived well with the metastasis [4, 7]. Our patient has been symptom-free for 1 year since the diagnosis of pulmonary metastasis, as reported by the family of the patient.

Immunohistochemistry and other newer diagnostic tests are commonly used for diagnosis, prognosis, and determining targeted therapies. Some recent studies in ameloblastoma indicate that BRAF mutations are frequently identified, particularly the BRAF V600E mutation, in some aggressive varieties of ameloblastoma. BRAF is a serine/threonine protein kinase that activates downstream signaling and increases cell proliferation and neoplastic transformation. This is considered an essential mutation in the pathogenesis of odontogenic tumors with an ameloblastomatous component. The incidence ranges from 43% to 82% of the studied tumors [8]. This is reported to be associated with multiple recurrences and even metastasis. BRAF inhibitors such as vemurafenib, dabrafenib, and sorafenib have significantly reduced the size in some metastatic and unresectable ameloblastoma cases [9, 10]. This may become the standard of care or a neoadjuvant therapy. One of the limitations in this case was that we could not do any mutation studies on the lung biopsy.

Presently, there are not enough studies to support or refute any treatment for METAM with absolute certainty. Physicians must tailor treatment for each patient, considering both tumor and host factors.

With this limited number of reported cases and variable tumor biology, it is also challenging to assess overall survival. Some authors report a median survival of 2 years with a mean disease-free interval of 14 years with pulmonary metastasis [11]. The heterogeneity of the tumor biology of METAM precludes the authors from confidently predicting survival outcomes.

## Conclusion

We present a case of metastasizing ameloblastoma in a female patient who had a 12-year history of recurrent ameloblastoma in the mandible. Metastasizing ameloblastoma is a rare variant that metastasizes but preserves benign histology. The long history and indolent nature of the metastasis may result in lack of identification or even misdiagnosis. A high degree of suspicion is mandatory for patients with locally aggressive ameloblastoma throughout their lifetime. It is essential to mention that most of these patients do not experience further deterioration. A better understanding of the underlying genetic mutations and involved pathways will open up newer therapeutic avenues. In future, tumoroid-based *in vitro* studies may be undertaken to understand these rare tumors.

## Supplementary Information

Additional file1

## Data Availability

The data that support the findings of this study are available on request from the corresponding author.

## Abbreviations

METAM: Metastasizing ameloblastoma
WHO: World Health Organization
CT: Computed tomography
